# Retraction: A comprehensive survey on the beliefs, perceptions, and clinical manifestations of pre and post Covid-19 vaccinations among physiotherapy students in the United Arab Emirates

**DOI:** 10.1371/journal.pone.0360878

**Published:** 2026-10-20

**Authors:** The PLOS One Editors

The *PLOS One* Editors retract this article [1] because it has come to light that the article’s peer review process was compromised. There are also concerns about compliance with PLOS policy on Manipulation of the Publication Process.

AH and SK did not agree with the retraction. SA, PK, and SG either did not respond directly or could not be reached.
